# The Tuberculosis Society

**Published:** 1914-06-20

**Authors:** Thompson Campbell

**Affiliations:** The Limes, Wakefield


### THE EDITOR'S LETTER-BOX.

### The Tuberculosis Society.

*To the Editor of* The Hospital.

Sir,—As you invite discussion, in your issue of June 6, regarding the association of the members of the medical profession who are devoting themselves to tuberculosis work, I desire to offer a friendly criticism of the constitution of the above society, with the purpose of assisting in adding to its strength. The secretary has been good enough to send me a copy of the rules, and it would appear that the administration is not sufficiently developed to meet the requirements of the whole country. I am informed that monthly meetings of the members are held in London; but such, I would point out, are obviously unsuited to the convenience of members in northern counties, and would offer no opportunity for discussion and the interchange of the fruits of experience in the case of officers remote from the Metropolis.

If a central organisation with an executive drawn from all parts of the country be not established, then we shall find a number of separate societies being formed which will lack the authority and prestige a united association would possess. The annual subscription of 5s. seems to me inadequate to meet the needs of a parent society, with the several branches necessary to permit members in grouped areas to meet regularly in order to fulfil the objects of such an association.

I have suggested elsewhere that a meeting of those interested might be held in Leeds on the evening of Wednesday, July 8, during the Annual Conference of the National Association for the Prevention of Consumption, and if members of the executive of the Tuberculosis Society were to attend, steps might be taken to expand the organisation and suggest amendments of the constitution in order that a Tuberculosis Society embracing the whole country might be evolved. Should such a meeting be supported I might intimate the fact in your issue of June 27 if you would kindly grant me space for the announcement, or otherwise communicate the information during the annual tuberculosis conference meetings.—I am, yours truly,

Thompson Campbell, M.D.

The Limes, Wakefield, June 12, 1914.

